# Endocrine Regulation on Bone by Thyroid

**DOI:** 10.3389/fendo.2022.873820

**Published:** 2022-04-05

**Authors:** Siyuan Zhu, Yidan Pang, Jun Xu, Xiaoyi Chen, Changqing Zhang, Bo Wu, Junjie Gao

**Affiliations:** ^1^Department of General Surgery, Shanghai Jiao Tong University Affiliated Sixth People’s Hospital, Shanghai, China; ^2^Department of Orthopaedic Surgery, Shanghai Jiao Tong University Affiliated Sixth People’s Hospital, Shanghai, China; ^3^Ningbo Institute of Life and Health Industry, University of Chinese Academy of Sciences, Ningbo, China

**Keywords:** thyroid, thyroid hormones, bone, bone homeostasis, thyroid diseases

## Abstract

**Background:**

As an endocrine organ, the thyroid acts on the entire body by secreting a series of hormones, and bone is one of the main target organs of the thyroid.

**Summary:**

This review highlights the roles of thyroid hormones and thyroid diseases in bone homeostasis.

**Conclusion:**

Thyroid hormones play significant roles in the growth and development of bone, and imbalance of thyroid hormones can impair bone homeostasis.

## 1 Introduction

As one of the most important endocrine organs, the thyroid regulates physiological processes by synthesizing and secreting calcitonin and thyroid hormones (THs). THs are essential for normal growth, differentiation and the physiological functions of various tissues (1), and thyroid-stimulating hormone (TSH) is secreted from the pituitary and regulates the synthesis and secretion of THs (2). Bone constitutes the skeletal structure that supports the human body and regulates calcium and phosphorus homeostasis (3). Normal bone remodeling involves a balance between osteoblasts mediated bone formation and osteoclasts mediated bone resorption (4). Currently, both thyroid diseases including hyperthyroidism and hypothyroidism, and bone diseases including osteoporosis are prevalent especially in women (5). Further, studies showed that hyperthyroidism causes osteoporosis and hypothyroidism impedes bone remodeling (6). This review summarized current studies about endocrine roles of thyroid on bone homeostasis.

## 2 The Thyroid, THs and TSH

The thyroid is an endocrine organ composed of thyroid follicular cells and interfollicular C cells. The thyroid mainly synthesizes calcitonin and THs including triiodothyronine (T3) and thyroxine (T4), which are regulated by the hypothalamus pituitary-thyroid axis (1). As functional units of the thyroid, thyroid follicles are surrounded by a single layer of epithelial cells (7). Each follicle is densely packed with blood vessels that play roles in the synthesis, preservation and secretion of T3/T4 into the bloodstream (8). Approximately 80% of T3 is produced by T4 transformation in peripheral tissues, whereas the remaining 20% is secreted directly from the thyroid (7). A lack of THs causes fatigue, constipation and weight gain, whereas excess THs can lead to cardiovascular diseases or increase osteoporosis (7). As an integral part of the hypothalamus pituitary-thyroid axis, TSH is closely related to THs. TSH promotes the growth and differentiation of the thyroid, as well as the secretion of THs. THs, in turn, regulate TSH through a negative feedback loop. Accordingly, under pathological conditions, enhanced negative feedback inhibits the pituitary function and results in decreased TSH secretion in hyperthyroidism, while weakened negative feedback inhibition results in increased TSH secretion in hypothyroidism to compensate for the body’s needs (2).

## 3 Bone Development and Bone Homeostasis

Bone is a rigid tissue that supports the human body. Bone cells, including osteoprogenitor cells, chondrocytes, osteoblasts, osteoclasts, and osteocytes, maintain bone homeostasis. Osteoprogenitor cells are bone stem cells, and these cells can differentiate into chondrocytes or osteoblasts under certain conditions. Chondrocytes participate in osteogenesis and assist joint movement. Osteoblasts conduct bone formation and subsequently differentiate into osteocytes. Endochondral and intramembranous ossification are two main ways of bone formation (9). During endochondral ossification, mesenchymal stem cells first differentiate into chondrocytes, and then chondrocytes undergo hypertrophy and apoptosis, after which cartilage lacunae forms. Thereafter, vascular vessels invade into cartilaginous tissue and then leads to the absorption of cartilage matrix mediated by osteoclasts and bone marrow lumen formation. Meanwhile, osteoblasts enter and attach to the bone marrow lumen to form bone tissue. While in intramembranous ossification, mesenchymal stem cells directly differentiate into osteoblasts, without the stage of chondrocytes (9, 10). Osteoclasts are involved in bone resorption, and jointly regulate bone remodeling with osteoblasts. As the most abundant bone cells, osteocytes are embedded into bone matrix and form dendritic network to regulate the balance between bone formation and resorption (11, 12). Osteocytes and osteoblasts play major roles in regulating osteoclasts by secreting receptor activator of nuclear factor kappa-B ligand (RANKL) and osteoprotegerin (OPG) (13). RANKL can bind with RANK on osteoclasts, which activates osteoclasts. The activation of osteoclasts depends on the ratio of RANKL and OPG, and slightly more OPG could bind with RANKL to prevent the binding between RANKL and RANK, and hinder osteoclast formation (14, 15). On the other hand, osteocytes specifically secrete sclerostin to inhibit bone formation of osteoblasts (16).

## 4 Effects of THs on Bone

### 4.1 TH Receptors (TRs) in Bone

TRs are widely distributed, and mainly in the nucleus and to a lesser degree in the cytoplasm (17). TRs are composed of three subtypes: TRα1, TRβ1 and TRβ2. TRα1 is mainly present in cardiac and skeletal muscles, TRβ1 mainly exists in the brain, kidney and liver, and TRβ2 is confined to the hypothalamus and pituitary, where the expression of thyrotropin releasing hormone (TRH) and TSH is inhibited (18–20). Both TRα1 and TRβ1 are expressed in bone, and TRα1 is approximately 10 times more abundant than TRβ1 (21). TRα1 plays a leading role when THs are at baseline concentrations, and TRβ1 rapidly responds to acute TH variations (22).

Moreover, general TRs also include receptors on the cell membrane, such as monocarboxylate transporter 8 (MCT8), MCT10, L-type amino acid transporter 1 (LAT1) and LAT2. The ability of MCT10 to transport T3 is better than that of MCT8, whereas MCT8 can better transport T4 than MCT10 (23). In global Mct8-knockout mice, increased numbers of osteoblasts and osteoclasts, accelerated bone turnover, and delayed bone mineralization were observed. While the absence of MCT8 in osteoclast progenitors (LysM^Cre^Mct8^f/f^) impaired osteoclastogenesis and subsequently impaired bone resorption. Interestingly, osteoprogenitor-specific MCT8-knockout mice (Osx^Cre^Mct8^f/f^) showed increased trabecular bone mass, indicating that MCT8 was a negative regulator of osteogenesis (24, 25).

### 4.2 THs Regulate Chondrocytes *via* hh-Parathyroid Hormone-Related Protein (PTHrP) Negative Feedback Loop

**Figure 1:**
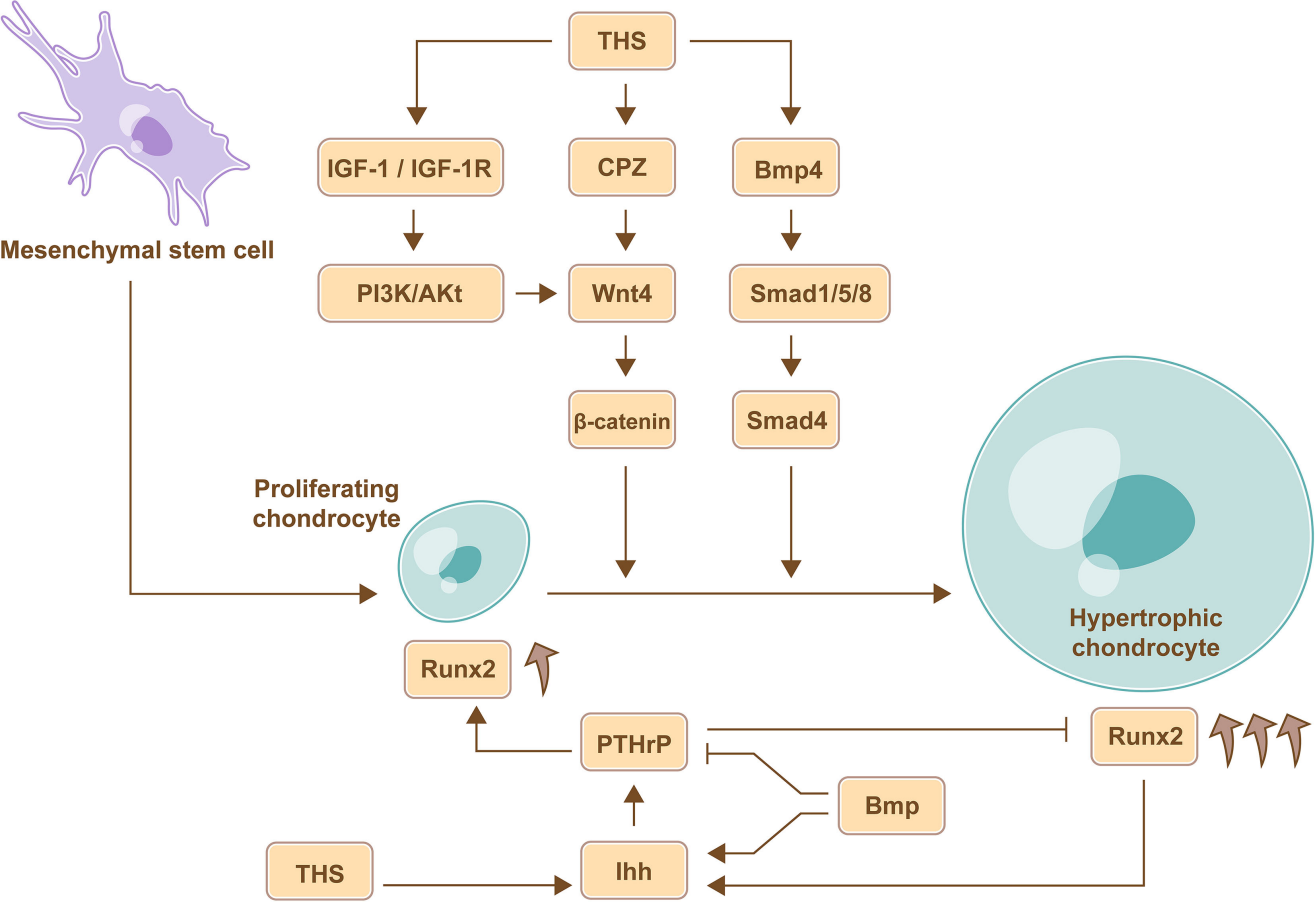
TH-mediate regulation of chondrocyte proliferation and differentiation. THs promote the differentiation of chondrocyte *via* Wnt signaling pathway, BMP signaling pathways, and IGF-1 signaling pathway. Furthermore, the expression of Ihh in pre-hypertrophic and hypertrophic chondrocytes induces the expression of PTHrP, which promotes chondrocyte proliferation and inhibits chondrocyte maturation through a negative feedback loop, and the site of hypertrophic chondrocytes determines the length of the bone, therefore, THs regulate the length of long bone by Ihh-PTHrP signaling pathway. Furthermore, BMP can increase the expression of Ihh and abrogates the partial inhibition of the maturation effects of PTHrP to regulate chondrocyte proliferation and maturation, and the increased expression of Runx2 in mature and differentiated chondrocytes is beneficial for stimulating chondrocyte proliferation mediated by Ihh.

THs regulate the process of chondrocyte proliferation and differentiation which is mediated by a series of crucial growth factors, including Indian hedgehog (Ihh), wingless/integrated (Wnt), insulin-like growth factor 1 (IGF-1) and bone morphogenetic protein (BMP) (26, 27) (**Figure 1**).

#### 4.2.1 THs Regulate Chondrocytes *via* Ihh-Parathyroid Hormone-Related Protein (PTHrP) Negative Feedback Loop

During endochondral ossification, chondrocytes go through three stages including proliferating chondrocytes, pre-hypertrophic chondrocytes and hypertrophic chondrocytes. Proliferating chondrocytes are around joints, and hypertrophic chondrocytes are formed by constantly moving forward of proliferating chondrocytes, the site of hypertrophic chondrocytes determines the length of the bone (28). Ihh is an intercellular signaling molecule in the Hh protein family (29) and is expressed in pre-hypertrophic and hypertrophic chondrocytes. The expression of Ihh in the tibial epiphyses could be increased by TH treatment (30). Subsequently, Ihh induces the expression of PTHrP, which promotes chondrocyte proliferation and inhibits chondrocyte maturation through a negative feedback loop. Therefore, TH regulates the length of long bone (31). Consistently, severe dwarfism and obviously declined rate of chondrocyte proliferation were observed in Ihh mutant mouse model (32). Therefore, THs regulate the length of long bone by controlling the location where the chondrocytes mature *via* Ihh-PTHrP negative feedback loop.

#### 4.2.2 THs Regulate Chondrocytes *via* Wnt Signaling Pathway

Carboxypeptidase Z (CPZ) is activated by THs and contains a cysteine-rich domain that binds to Wnt4; CPZ promotes the removal of C-terminal amino acids from Wnt4 and then enhances Wnt4 activity (33). The expression of Wnt4 favors the accumulation of stabilized β-catenin, which promotes chondrocyte differentiation, and the expression of Runx2, which is beneficial for stimulating chondrocyte proliferation mediated by Ihh (34). Therefore, THs promote chondrocyte proliferation and differentiation *via* Wnt signaling pathway.

#### 4.2.3 THs Regulate Chondrocytes *via* IGF-1 Signaling Pathway

In rat model, the expression of IGF-1 receptor (IGF-1R) and the chondrocyte differentiation markers including collagen X and alkaline phosphatase (ALP) activity in growth plate cells were significantly upregulated under the T3 treatment, and subsequent study further demonstrated that THs stimulate chondrocyte differentiation by upregulating Wnt4 expression and accumulation of β-catenin *via* IGF-1/PI3K/Akt signaling pathway (35).

#### 4.2.4 THs Regulate Chondrocytes *via* BMP/Smad Signaling Pathway

In the upper portion of the embryonic chick sternum, the expression of BMP4 in chondrocytes was increased following T3 treatment. BMP belongs to the TGF-β superfamily, and the Smad protein family mediates signal transduction of different TGF-β family members. BMP promotes Smad 1/5/8 phosphorylation and then coactivates Smad 4 and induces the expression of chondrocyte differentiation markers, such as collagen X (36). Further, BMP increases the expression of Ihh and abrogates the partial inhibition of the maturation effects of PTHrP to regulate chondrocyte proliferation and maturation (37).

### 4.3 Effects of THs on Osteoblasts

#### 4.3.1 The Function of Type 2 Deiodinase (DIO2) in Osteoblasts

**Figure 2:**
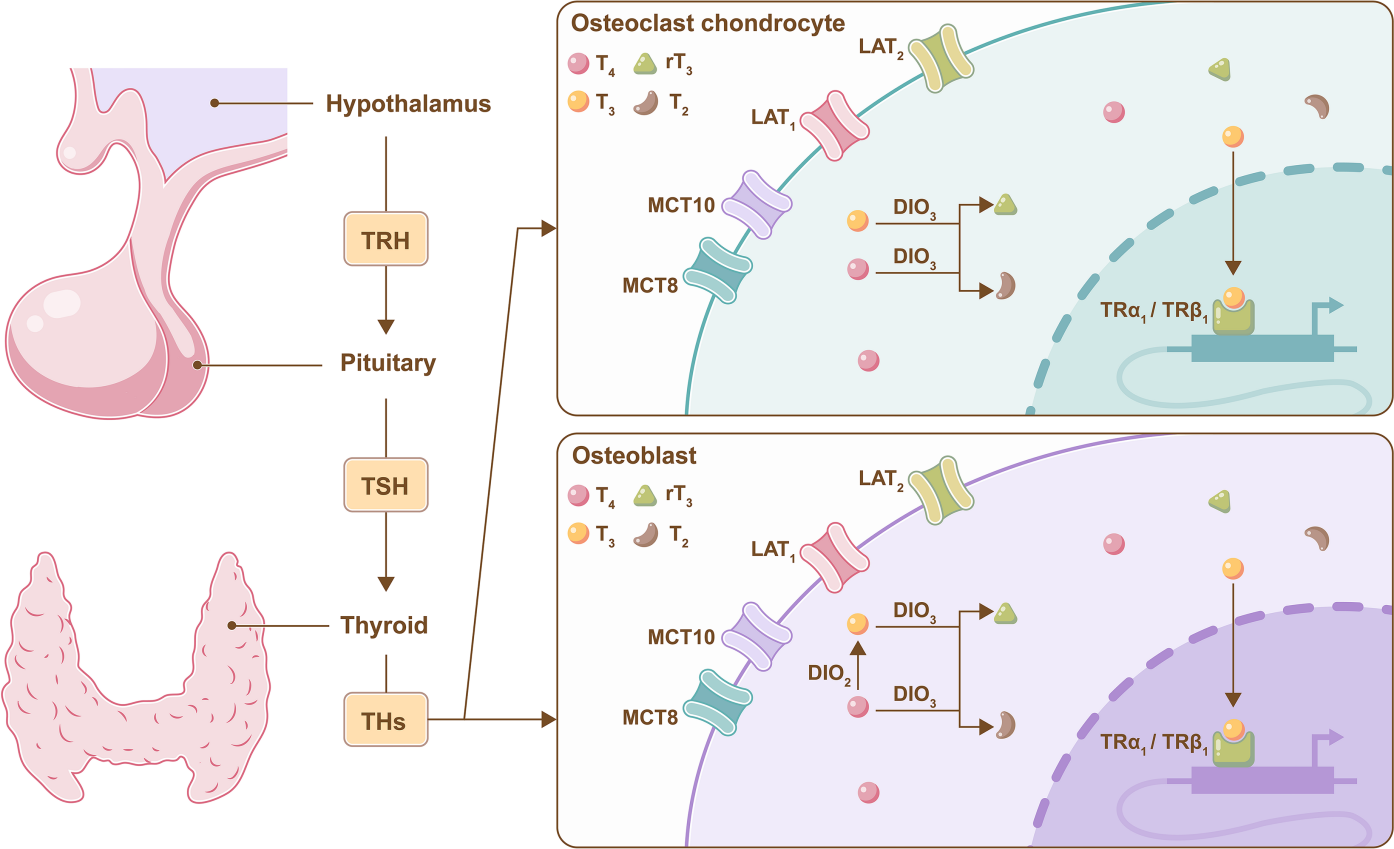
Metabolism of THs in bone cells. A brief illustration of the negative feedback regulation of TH mediated by the hypothalamus pituitary-thyroid axis, and DIO2 converts T4 to bioactive T3 in osteoblasts, and DIO3 converts both T3 and T4 to T2 and rT3 in osteoclasts, chondrocytes and osteoblasts.

Deiodinases (DIOs) are present in target tissues and can amplify or terminate TH signaling through DIO2 and DIO3 (38). DIO2 converts T4 to bioactive T3, and DIO3 converts both T3 and T4 to diiodothyronine (T2) and reverse T3 (rT3); the latter two are dysfunctional proteins (39). DIO2 is found in mature primary osteoblasts in bone, while DIO3 is present in chondrocytes, osteoblasts and osteoclasts (40). Knockout DIO2 in osteoblasts exhibited increased bone mineralization, low tough femurs and fracture tendency, which was consistent with the clinical manifestations of hypothyroidism in bone tissue (41, 42). Therefore, DIO2 is essential for proper osteoblast function (**Figure 2**).

#### 4.3.2 THs Regulate Osteoblasts *via* Wnt Signaling

THs inhibit the differentiation of osteoblasts by inhibiting Wnt/β-catenin signaling pathway. T3 increased reporter gene activity mediated by TRα1 and TRβ1, whereas T3 inhibited β-catenin pathway reporter gene activity in UMR106 cells that were co-transfected with TRα1 or TRβ1. In the absence of TRs or T3, β-catenin pathway reporter gene activity was not affected, and a similar outcome was observed in osteoblastic MC3T3 cells. Therefore, T3 inhibits the Wnt/β-catenin signaling pathway in osteoblasts (43).

#### 4.3.3 THs Regulate Osteoblasts *via* BMP Signaling

THs promote the differentiation of osteoblasts by BMP/Smad signaling pathway. The expression of differentiation markers was increased, and Smad1/5/8 phosphorylation was mediated by BMP signaling when osteoblasts were treated with T3. This finding suggested that THs could promote osteoblast differentiation *via* the BMP/Smad signaling pathway (44). C2C12 myoblasts were transfected with a BMP/Smad-specific reporter construct and treated with Bmp2 or Wnt3a ligands, and the results showed that not only Bmp2 but also Wnt3a enhanced BMP/Smad activity. Furthermore, the overexpression of β-catenin could activate Bmp2 overexpression. This finding showed that the Wnt/β-catenin signaling pathway stimulated the Bmp2/Smad signaling pathway in osteoblasts. Furthermore, Bmp2/Smad signaling pathways could also regulate the Wnt/β-catenin signaling pathway; these pathways interacted with each other and regulated target gene expression by forming a transcriptional complex (Smad bound with β-catenin) in osteoblasts (45).

#### 4.3.4 THs Regulate Osteoblasts *via* IGF-1 Signaling

**Figure 3:**
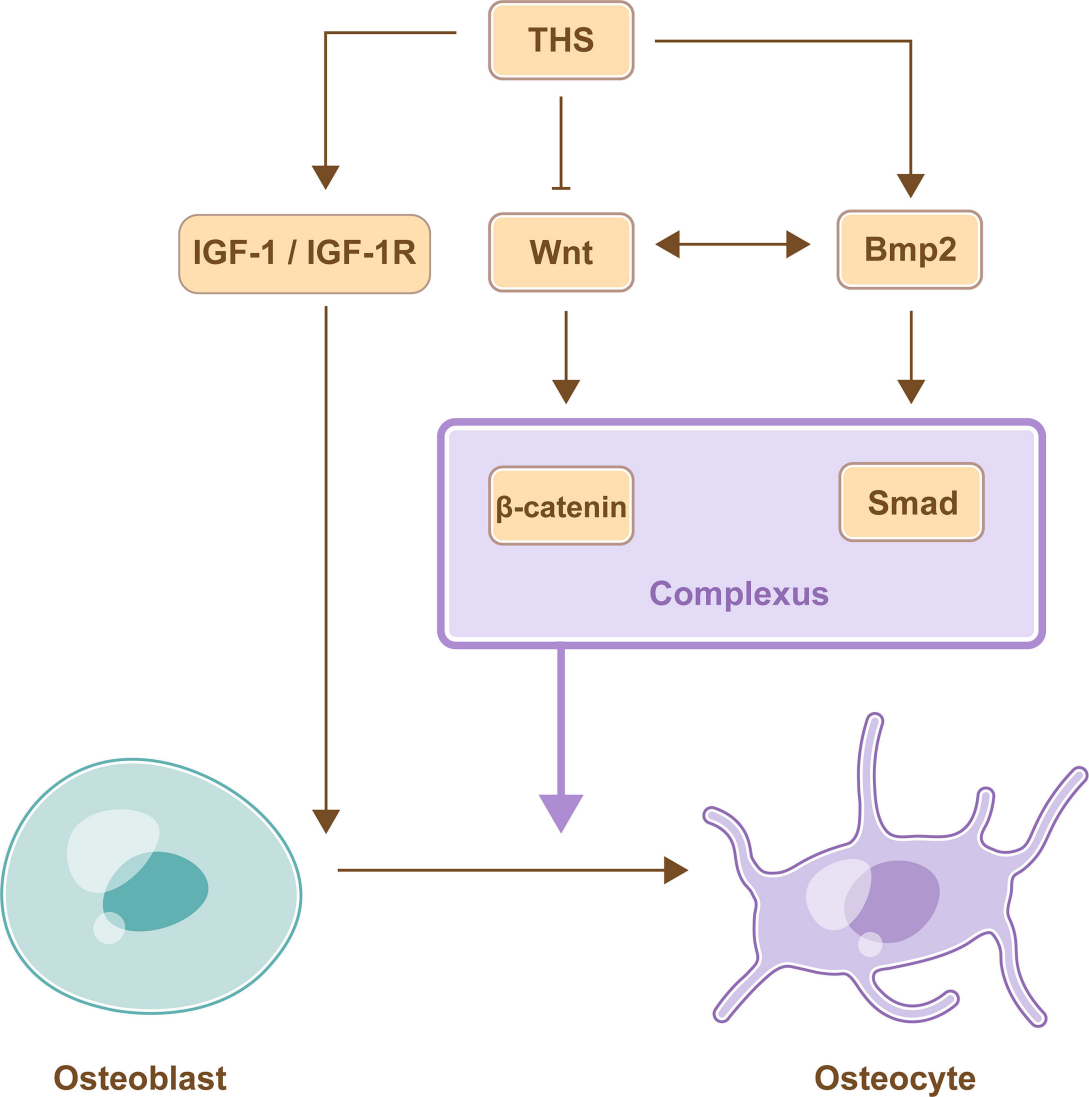
TH-mediate regulation of osteoblast differentiation. THs promote the differentiation of osteoblasts by activating IGF-1 signaling pathway and BMP signaling pathway, while THs inhibit the differentiation of osteoblasts by inhibiting Wnt/β-catenin signaling pathway. Furthermore, BMP signaling pathway and Wnt signaling pathway interact with each other and promote differentiation of osteoblast by forming a transcriptional complex.

THs promote the differentiation of osteoblasts by IGF-1 signaling pathway. IGF-1 mRNA levels were increased in MC3T3-E1 cells after treatment with THs, and the increased levels were positively correlated with TH concentrations. Furthermore, their metabolites, including T2 and rT3, could also promote the increase in IGF-1 mRNA levels (46) (**Figure 3**).

#### 4.3.5 THs Regulate the Expression of Osteocalcin (Ocn)

Ocn is produced exclusively by osteoblasts, and increased bone formation in both trabecular and cortical bone in Ocn knockout (Ocn^-/-^) mice, indicating that the function of osteoblast increased. Further, the increased osteoclast number in Ocn^-/-^ mice, indicating increased osteoclast function. Therefore, Ocn is a negative regulator of bone formation and resorption (47). Study showed that triiodothyronine (T3) promotes Ocn synthesis in osteoblast−like cells *in vitro* (48).

### 4.4 The Role of THs in Osteoclasts

Osteoclasts are derived from hematopoietic progenitors and promote bone resorption (49). Studies showed that T3 increased the RANKL/OPG ratio in the femur in wild-type mice but not in β2-adrenergic receptor (AR)^-/-^ mice, indicated that T3 activated osteoclasts function through the β2-AR pathway in bone (50). In addition, thyrotoxicosis impaired bone mineral density (BMD) in WT mice, and the stimulative effect to bone resorption was more stronger than bone formation (51), however, BMD was not significantly decreased in response to the supraphysiological dose of T3 in α2A/C-AR double knockout mice, suggesting that α2-AR mediated T3-induced bone resorption (52). On the other hand, the expression of c-Fos protein increased in osteoclast progenitor cells after treatment with THs, and inhibiting the expression of c-Fos protein by antisense oligodeoxynucleotides (as-ODN) inhibited the ability of THs to induce the formation of osteoclasts, therefore, THs promoted the differentiation of osteoclasts, which was at least partly mediated by the upregulation of c-Fos protein in osteoclast precursor cells (53). In conclusion, THs mainly promote the developing of growing bone and stimulate remodeling of mature bone (**Table 1**).

**Table 1:** Thyroid effect on the components of the bone tissue.

	Thyroid effect on chondrocyte	Thyroid effect on osteoblast	Thyroid effect on osteoclast
***In vivo***	Stimulate the expression of collagen X and ALP	Stimulate the expression of osteocalcin and bone formation	Stimulate the expression of tartrate-resistant acid phosphatase (TRAP) and osteoclast formation
***In vitro***	Promote growth plate chondrocyte proliferation and terminal differentiation	Stimulate osteoblast differentiation and inhibit osteoblast proliferation	Stimulate osteoclast differentiation
**Human data**	Promote endochondral bone formation	Participate in bone mass maintenance	Participate in bone mass maintenance

## 5 Thyroid Dysfunction (Hyperthyroidism and Hypothyroidism) Acts on Bone

Hyperthyroidism, which is a form of thyrotoxicosis, is characterized by high TH serum levels and low TSH serum levels (54), while subclinical hyperthyroidism is a state of low TSH serum levels with normal T3 and T4 serum levels (1). Hypothyroidism and subclinical hypothyroidism are just the opposite. Hypothyroidism is characterized by low TH serum levels and high TSH serum levels (55), and subclinical hypothyroidism is a state of high TSH serum levels with normal T3 and T4 serum levels (56). Individuals with hyperthyroidism or hypothyroidism could experience bone loss and low BMD (57) and are at risk of osteoporosis and even fracture (58), but BMD back to normal after returning to the euthyroidism state (59, 60) (**Table 2**).

**Table 2:** Thyroid dysfunction acts on bone.

Thyroid disease	Pathogenesis	Common clinical manifestation	Effect on bone	Treatment outcome
Hyperthyroidism in child	Graves’ disease	Hoarseness and difficulty concentrating	1.Premature bone formation leading to short stature; 2.Craniosynostosis.	Ameliorate symptoms, and increase BMD
Hyperthyroidism in adult	1.Graves’ disease; 2.Toxic multinodular goiter; 3.Toxic adenoma.	Fatigue, anxiety, palpitation, weight loss, heat intolerance, tachycardia, tremor, poor concentration, goiter	Stimulate the differentiation of osteoblasts and osteoclasts, promote more bone resorption than bone formation, low BMD and osteoporosis even fractures	Ameliorate symptoms, and increase BMD
Hyperthyroidism in aging	1.Graves’ disease; 2.Toxic multinodular goiter; 3.Use of amiodarone or iodinated contrast agents.	1.Neurocognitive changes; 2.Cardiovascular disease such as atrial fibrillation; 3.Weight loss.	Severe osteoporosis	Ameliorate symptoms, and increase BMD
Hypothyroidism in child	1.Autoimmune disease; 2.Iodine deficiency associated with goiter; 3.Congenital hypothyroidism: thyroid agenesis and dyshormonogenesis, panhypopituitarism.	1.Nonspecific symptoms such as prolonged jaundice, feeding difficulties, lethargy, hoarse cry and hypotonia in newborn; 2.Higher risk for obesity and metabolic syndrome and cardiovascular disease in child.	1.Delayed skeletal development, growth retardation, short stature; 2.Delayed closure of the fontanelles, persistently patent skull sutures.	Reach a height rapidly and nonspecific symptoms are relieved
Hypothyroidism in adult	1.Autoimmune disease; 2.Invasive or compressive lesions: Pituitary macroadenomas; 3.Iatrogenic factors and drug-induced hypothyroidism.	Nonspecific symptoms such as fatigue, weight gain, constipation, dry hair, dry skin	Reduced bone remodeling and increased bone mass, osteosclerosis and fracture risk	Levothyroxine replacement therapy leads to transient bone loss and increased fracture risk, and BMD returns to normal after a time
Hypothyroidism in aging	1.Autoimmune disease; 2.Iatrogenic factors and drug-induced hypothyroidism.	Symptoms and signs are mild or even absent, such as high cholesterol, diastolic hypertension, constipation, heart failure, fatigue, depression, forgetfulness	/	Improves clinical symptoms associated with hypothyroidism
TRα1/TRβ mutation-mediated TH resistance	/	1.Similar to hypothyroidism (TRα1 mutation); 2.Similar to hyperthyroidism (TRβ mutation).	1.Reduced bone remodeling and accumulated bone mass (TRα1 mutation); 2.Low BMD and osteoporosis even fractures (TRβ mutation).	1.Similar to hypothyroidism (TRα1 mutation); 2.Similar to hyperthyroidism (TRβ mutation).
Bone metastases of thyroid cancer	/	Bone destruction and bone hyperplasia	Bone destruction and bone hyperplasia	According to the results of thyroid cancer treatment

### 5.1 Impact on Children

Hypothyroidism in children impairs both endochondral and intramembranous ossification, which manifest as delayed bone development, short stature, delayed closure of fontanelles and persistently patent skull sutures. According to updated consensus guidelines, the incidence of primary congenital hypothyroidism was approximately 1 in 2500, and LT4 was recommended as a therapy (61). TH replacement therapy could contribute to rapid growth, even if the final height may not match that of normal children (62). Cessation of growth is common in children with fearful hypothyroidism (63, 64). However, bone health is not impaired in children despite maintaining the state of subclinical hypothyroidism over time (65).

**Figure 4:**
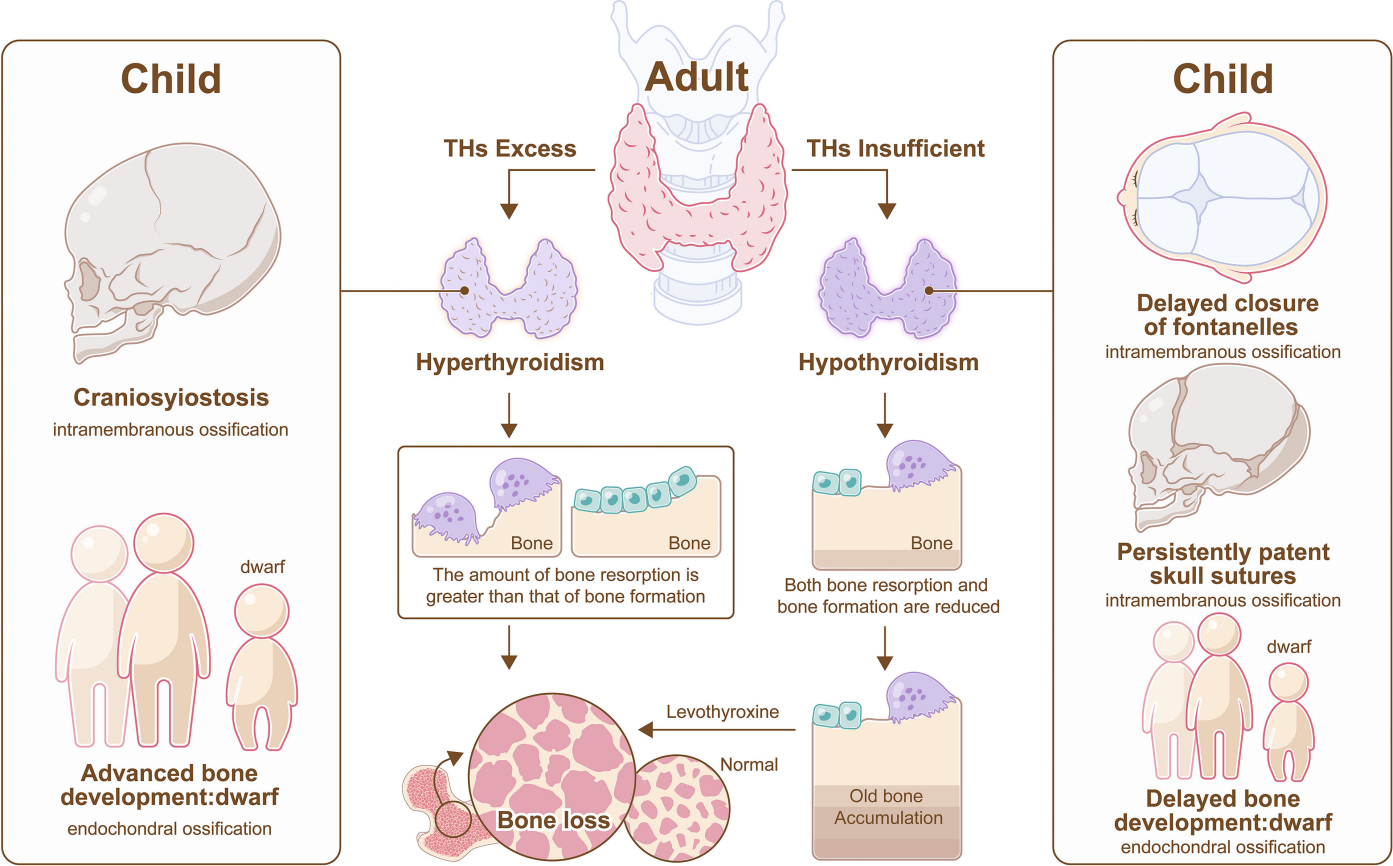
Hyperthyroidism and hypothyroidism on bone. In adult, hyperthyroidism promotes more bone resorption than bone formation, resulting in bone loss, and hypothyroidism impedes bone remodeling, resulting in old bone accumulation, however, bone loss also appears in hypothyroidism after the administration of LT4. In child, hypothyroidism impairs both endochondral and intramembranous ossification, which manifest as delayed closure of fontanelles, persistently patent skull sutures, and short stature, and hyperthyroidism results in craniosynostosis and below-average height.

On the other hand, children with thyrotoxicosis have below-average height and craniosynostosis (66), and an elevated free T4 serum concentration may associate with low BMD (67) (**Figure 4**).

### 5.2 Impact on Adults

#### 5.2.1 Clinical Thyroid Dysfunction

Hyperthyroidism can promote more bone resorption than bone formation, resulting in osteoporosis (51). Thyroid surgery is promising to decrease fracture risk in patients with hyperthyroidism (58), and treatment with antithyroid drugs can achieve a similar effect because the synthesis of THs is catalyzed by thyroid peroxidase, which can be inhibited by antithyroid drugs (55).

Hyperthyroidism induces the expression of sclerostin, subsequently leading to osteoporosis, but a dramatic decline in serum sclerostin occurs after treatment with drugs. Therefore, sclerostin may be a potential therapeutic target in hyperthyroidism related osteoporosis (68). On the other hand, excessive activation of BMP and Wnt pathway were observed in hyperthyroidism, indicating that BMP or Wnt pathway may be other therapeutic targets for hyperthyroidism-induced bone loss (44, 69).

In patients with hypothyroidism, bone turnover was impaired, and bone mass increased, which could cause a transient decrease in BMD after the administration of synthetic levothyroxine (LT4) (55). In addition to bone tissue, joints are also affected in hypothyroidism, which is characterized by arthralgias, arthritis and aseptic necrosis (70).

#### 5.2.2 Subclinical Thyroid Dysfunction

Women with subclinical hyperthyroidism have reduced BMD in the hip and femoral neck, especially those with TSH levels less than 0.10 mIU/L, which can increase the risk of fracture (71, 72). On the other hand, studies indicate that most subclinical hypothyroidism does not require treatment and could even reduce the incidence of osteoporosis in postmenopausal women (51, 56). Studies have shown that subclinical hypothyroidism may lead to cardiovascular disease, and a small proportion of patients with subclinical hypothyroidism take LT4 to prevent cardiovascular disease (73). However, LT4 therapy enhances bone turnover and causes bone loss (74). In this context, whether LT4 treatment is used depends on the balance between the benefits and risks.

### 5.3 Impact on Aging

Hypothyroidism is the most common thyroid disease among the elderly, with insidious onset and slow progression. Thyroid hypofunction is conducive to prolonging life expectancy. However, the elderly will be more prone to disability, cognitive impairment, shortened life expectancy and other adverse events if thyroid function reaches a certain low level without timely treatment. But the link between hypothyroidism and bone in the elderly is not well established (75). In addition, the incidence of hyperthyroidism is also high, but the clinical manifestations are not typical, such as neurocognitive changes, cardiovascular diseases and unexplained weight loss. In addition, hyperthyroidism aggravates bone loss in the elderly, leading to more serious osteoporosis (76).

### 5.4 Bone Manifestations of Thyroid Hormone Resistance (RTH)

RTH is defined as abroad tissue hyporesponsiveness to THs with normal or raised TSH or TH serum concentrations, and RTH is usually caused by TRα1 or TRβ1 mutations (77). TRβ1 mutation is more common than TRα1 mutation in RTH, and increased TH serum concentrations associated with TRβ1 mutation increase TRα1 activity and cause hyperthyroidism (78). TRβ1^+/-^ mice had almost normal phenotypes, while fearful osteoporosis developed in TRβ1^-/-^ mice, bone mass was greatly decreased (79, 80). On the other hand, patients with TRα1 mutations exhibit RTH and delayed bone development. The severity of TRα1 mutations depends on the mutation location and number. Manifestations in patients with missense mutations are always not as severe as those in patients with frameshift and nonsense mutations (21, 77).

## 6 Thyroid Cancer and Bone Metastasis (BM)

Thyroid cancer is the most common endocrine gland cancer and includes papillary thyroid cancer (PTC), follicular thyroid cancer (FTC), medullary thyroid cancer (MTC), and anaplastic thyroid cancer (ATC). PTC and FTC are differentiated thyroid cancers (DTCs), which account for 85% to 90% of all thyroid cancers. Most have a good prognosis, but the occurrence of distant metastases, including BM leads to decreased survival in a minority of cases, and the 10-year survival rate for most patients with BM is less than 50% (81). MTC is a neuroendocrine tumor that secretes calcitonin and originates from interfollicular C cells. ATC has an extremely low survival rate, particularly when accompanied by BM (82). DTCs have fewer distant metastases, and their BM are fewer than that of ATC and MTC. BM consist of osteolytic metastases, osteoblastic metastases, and mixed metastases. Osteolytic BM account for the majority of BM, and spine is the most common site of BM (83, 84).

### 6.1 Osteolytic Metastases in Thyroid Cancer

The RANKL serum concentration was higher in thyroid cancer patients with BM than in those without metastasis or with lung metastasis alone, and the outcomes that metformin inhibited ATC tumor growth in BM by inhibiting osteoblastic RANKL production and osteoclast differentiation indicated BM of thyroid cancer were at least partly mediated by increasing the level of RANKL in osteoblasts followed by activating osteoclast differentiation and causing bone resorption (84).

### 6.2 Osteogenic Behavior and Other Forms of Calcification in Thyroid Cancer

Calcification consists of osteogenesis, psammoma bodies and stromal calcification, the latter two often occur in PTC and MTC. BMP1 was expressed at much higher levels in PTC with psammoma bodies or stromal calcification (85). However, it was found that expression of BMP9 was not significantly elevated in bone formation of BM while the increased ALK1 (receptor protein kinase of BMP) could give a reasonable explanation to osteogenesis behavior (86). In contrast to other thyroid cancers, MTC can secrete calcitonin, the receptor of which is only expressed in osteoclasts. Treatment with calcitonin increased the expression of Wnt10b and ALP in osteoclasts and osteoblasts, respectively. Furthermore, pretreatment with the Wnt secretion inhibitor C59 further increased the expression of Wnt10b in osteoclasts and reduced the expression of ALP in osteoblasts. Therefore, MTC secretes calcitonin and induces bone formation by increasing the expression and secretion of Wnt10b in osteoclasts (87).

### 6.3 Therapy and Its Influence on Bone

In DTC patients with BM, treatment aims to control pain and local tumor development by radioiodine therapy, pharmacologic therapy or surgical treatments (88). Radioiodine therapy alone or combined with other treatments can dramatically increase overall survival (89). Suppressive LT4 therapy is a method to inhibit the concentration of TSH by negative feedback of exogenously increased THs to reduce the tumor recurrence rate. Most patients with DTC need lifelong medication, which may affect bone metabolism. However, suppressive LT4 therapy (TSH ≤0.4 mIU/L) not only fails to lower tumor recurrence but also causes bone toxicity and osteoporosis in patients without a high recurrence risk of DTC (90). Regardless, bone resorption markers return to normal with LT4 withdrawal (91). In addition, less than 2.6 μg/kg LT4 may not influence bone metabolism in DTC patients with normal estrogen (92), as estrogen plays important roles in protecting against bone loss by promoting OPG expression and enhancing osteoblast activity (93). Therefore, proper LT4 therapy has a marginal effect on bone degradation (94, 95). Thyroid related diseases are classified in **Table 2**.

## 7 The Role of TSH in Bone

TSH receptors are distributed in not only the thyroid but also osteoclasts and osteoblasts, and TSH affects bone homeostasis independent of THs (96). TSH shows inhibitory effects on bone resorption and active effects on osteogenesis (97, 98).

Research showed that as the concentration of rhTSH increased, the formation of osteoclasts was inhibited (99), and the mechanism by which TSH inhibits osteoclastogenesis was increasing the expression of OPG and decreasing the expression of RANKL on osteoblasts (100). Furthermore, TSH suppressed the expression of tumor necrosis factor α (TNFα) which inhibits osteoclastogenesis and the quantity of osteoclasts (101, 102). On the other hand, adding TSH to osteogenic medium promoted the expression of osteogenic markers and significantly increased the level of Wnt5a in embryonic stem cells (ESCs) (103).

Hyperthyroidism is characterized by excessive THs and low TSH (104), which is consistent with the discovery that patients with hyperthyroidism have reduced BMD due to a lack of protection from TSH (105). However, TSH-βv, which is a TSH-β subunit originating from macrophages in mice, was increased to compensate for the limited bone protection caused by reduced TSH (96, 100), and this finding was consistent with a study showing that the osteogenic markers in adult femurs were inhibited by anti-TSH-β (106).

## 8 Conclusion and Perspective

Homeostasis of thyroid are indispensable in the normal growth and development of bone, and the regulatory effect and mechanism of the thyroid on each type of bone cell as well as bone diseases were reviewed in detail.

**Figure 5:**
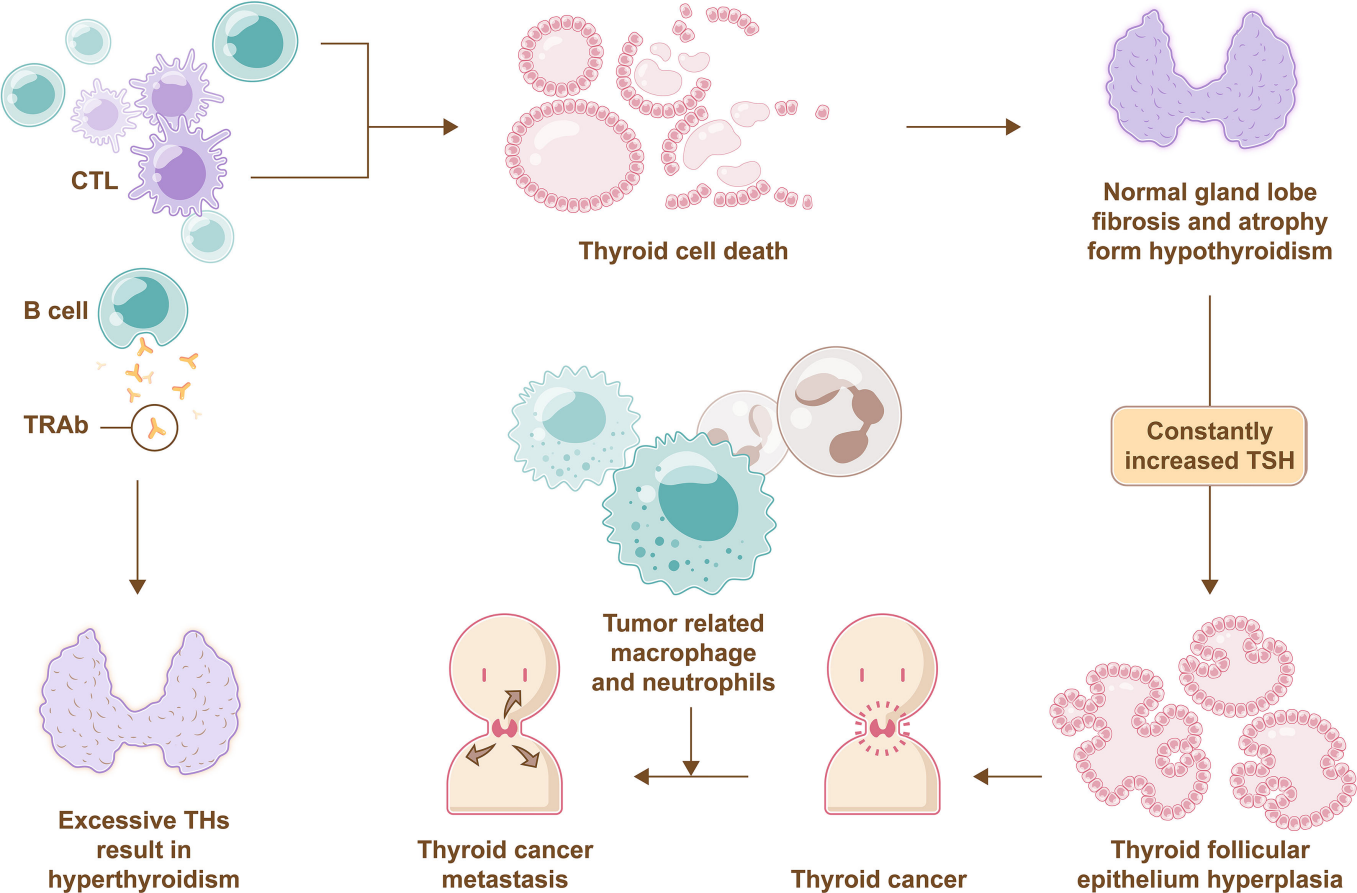
Immune imbalance in thyroid disease. Normal thyroid cells die due to attacks of B cells and CTL, and gland lobes undergo fibrosis and atrophy, which subsequently leads to hypothyroidism that promotes TSH secretion through negative feedback, then excessive TSH lead to pathological hyperplasia of thyroid folicullar epithelium followed by inducing thyroid cancer. Muchmore, tumor-related macrophages and neutrophils in patients with thyroid cancer can promote invasion and metastases of tumor cells. Furthermore, thyrotropin receptor antibodies (TRAb) produced by B cells stimulate TH production and result in hyperthyroidism.

In addition, more attentions should also be paid on the roles of bone marrow in various thyroid diseases. Because of the active hemopoietic ability of bone marrow, studies of immune cells from bone marrow and the thyroid are gradually being carried out. Bone marrow consists of hematopoietic stem cells, which are the ancestors of immune cells such as lymphocytes, granulocytes, and mononuclear macrophages. Immune cell imbalance is closely related to autoimmune thyroid disorders. In thyroiditis, the immune balance is disrupted, and the thyroid gland is gradually infiltrated with lymphocytes, including B cells and cytotoxic T cells. Eventually, normal thyroid cells are attacked and die, and gland lobes undergo fibrosis and atrophy, which subsequently leads to hypothyroidism and thyroid cancer (107). In Graves’ disease, elevated thyroid-stimulating immunoglobulins (also called thyrotropin receptor antibodies) produced by B cells stimulate TH production and result in hyperthyroidism. Specific autoantibodies for cancer antigens, tumor-related macrophages and neutrophils in patients with thyroid cancer could promote invasion and metastases of tumor cells and disrupt immune monitoring (108, 109) (**Figure 5**). Currently, autoimmune thyroid disorders have been defined as independent risk factors for thyroid cancer, even the opinion that the origin of the cancer is connected with a wide and severe immune stimulus has been put forward (110).

While bone marrow stem cells (BMSCs), which are another group of cells that can be isolated from bone marrow, have also attracted much attention in the field of tissue repair and regenerative medicine. BMSCs can differentiate into various kinds of cells. However, little research focus on the potential role of BMSCs to differentiate into thyroid follicular cells *in vitro*. Interestingly, the ATDC-5 cell line, a type of chondrogenic cell line, can express thyroglobulin (Tg), which is a thyroid-specific protein that is regulated by the transcription factor TTF-1 (111). Thus, further research is worth to determine the potential for bone-derived stem cells to differentiate into thyroid cells.

## Author Contributions

JG, BW, and CZ conceived and designed the review. SZ and YP wrote the manuscript. JX and XC provided suggestions. All authors contributed to the article and approved the submitted version.

## Funding

This study was done with the support of National Natural Science Foundation of China (82002339) and Shanghai Sixth People’s Hospital Scientific Research Foundation.

## Conflict of Interest

The authors declare that the research was conducted in the absence of any commercial or financial relationships that could be construed as a potential conflict of interest.

## Publisher’s Note

All claims expressed in this article are solely those of the authors and do not necessarily represent those of their affiliated organizations, or those of the publisher, the editors and the reviewers. Any product that may be evaluated in this article, or claim that may be made by its manufacturer, is not guaranteed or endorsed by the publisher.

## References

[1] Klonisch T Hoang-Vu C Hombach-Klonisch S. Thyroid Stem Cells and Cancer. Thyroid (2009) 19(12):1303–15. doi: 10.1089/thy.2009.1604 20001714

[2] Kopp P. The TSH Receptor and its Role in Thyroid Disease. Cell Mol Life Sci: CMLS (2001) 58(9):1301–22. doi: 10.1007/PL00000941 PMC11337400 11577986

[3] Oldknow KJ MacRae VE Farquharson C. Endocrine Role of Bone: Recent and Emerging Perspectives Beyond Osteocalcin. J Endocrinol (2015) 225(1):R1–19. doi: 10.1530/JOE-14-0584 25655764

[4] Armas LAG Recker RR. Pathophysiology of Osteoporosis: New Mechanistic Insights. Endocrinol Metab Clin North Am (2012) 41(3):475–86. doi: 10.1016/j.ecl.2012.04.006 22877425

[5] Estell EG Rosen CJ. Emerging Insights Into the Comparative Effectiveness of Anabolic Therapies for Osteoporosis. Nat Rev Endocrinol (2021) 17(1):31–46. doi: 10.1038/s41574-020-00426-5 33149262

[6] Vandenbroucke A Luyten FP Flamaing J Gielen E. Pharmacological Treatment of Osteoporosis in the Oldest Old. Clin Interventions Aging (2017) 12:1065–77. doi: 10.2147/CIA.S131023 PMC5505539 28740372

[7] Ogundipe VML Groen AH Hosper N Nagle PWK Hess J Faber H. Generation and Differentiation of Adult Tissue-Derived Human Thyroid Organoids. Stem Cell Rep (2021) 16(4):913–25. doi: 10.1016/j.stemcr.2021.02.011 PMC8072035 33711265

[8] Villacorte M Delmarcelle A-S Lernoux M Bouquet M Lemoine P Bolsée J. Thyroid Follicle Development Requires Smad1/5- and Endothelial Cell-Dependent Basement Membrane Assembly. Dev (Cambridge England) (2016) 143(11):1958–70. doi: 10.1242/dev.134171 PMC4920163 27068110

[9] Wojcicka A Bassett JHD Williams GR. Mechanisms of Action of Thyroid Hormones in the Skeleton. Biochim Biophys Acta (2013) 1830(7):3979–86. doi: 10.1016/j.bbagen.2012.05.005 22634735

[10] Hinton RJ Jing Y Jing J Feng JQ. Roles of Chondrocytes in Endochondral Bone Formation and Fracture Repair. J Dental Res (2017) 96(1):23–30. doi: 10.1177/0022034516668321 PMC5347428 27664203

[11] Williams GR Bassett JHD. Thyroid Diseases and Bone Health. J Endocrinol Invest (2018) 41(1):99–109. doi: 10.1007/s40618-017-0753-4 28853052 PMC5754375

[12] Buck DW 2nd Dumanian GA. Bone Biology and Physiology: Part I. The Fundamentals. Plast Reconstr Surg (2012) 129(6):1314–20. doi: 10.1097/PRS.0b013e31824eca94 22634648

[13] Nakashima T Hayashi M Fukunaga T Kurata K Oh-Hora M Feng JQ. Evidence for Osteocyte Regulation of Bone Homeostasis Through RANKL Expression. Nat Med (2011) 17(10):1231–4. doi: 10.1038/nm.2452 21909105

[14] Guo J Ren R Sun K Yao X Lin J Wang G. PERK Controls Bone Homeostasis Through the Regulation of Osteoclast Differentiation and Function. Cell Death Dis (2020) 11(10):847. doi: 10.1038/s41419-020-03046-z 33051453 PMC7554039

[15] Han Y You X Xing W Zhang Z Zou W. Paracrine and Endocrine Actions of Bone-the Functions of Secretory Proteins From Osteoblasts, Osteocytes, and Osteoclasts. Bone Res (2018) 6:16. doi: 10.1038/s41413-018-0019-6 29844945 PMC5967329

[16] Robling AG Bonewald LF. The Osteocyte: New Insights. Annu Rev Physiol (2020) 82:485–506. doi: 10.1146/annurev-physiol-021119-034332 32040934 PMC8274561

[17] Cheng S-Y Leonard JL Davis PJ. Molecular Aspects of Thyroid Hormone Actions. Endocrine Rev (2010) 31(2):139–70. doi: 10.1210/er.2009-0007 PMC2852208 20051527

[18] Singh BK Sinha RA Zhou J Tripathi M Ohba K Wang M-E. Hepatic FOXO1 Target Genes Are Co-Regulated by Thyroid Hormone via RICTOR Protein Deacetylation and MTORC2-AKT Protein Inhibition. J Biol Chem (2016) 291(1):198–214. doi: 10.1074/jbc.M115.668673 26453307 PMC4697156

[19] Gogakos AI Duncan Bassett JH Williams GR. Thyroid and Bone. Arch Biochem Biophys (2010) 503(1):129–36. doi: 10.1016/j.abb.2010.06.021 20599658

[20] Ambrosio R Damiano V Sibilio A De Stefano MA Avvedimento VE Salvatore D. Epigenetic Control of Type 2 and 3 Deiodinases in Myogenesis: Role of Lysine-Specific Demethylase Enzyme and Foxo3. Nucleic Acids Res (2013) 41(6):3551–62. doi: 10.1093/nar/gkt065 PMC3616708 23396445

[21] van Gucht ALM Moran C Meima ME Visser WE Chatterjee K Visser TJ. Resistance to Thyroid Hormone Due to Heterozygous Mutations in Thyroid Hormone Receptor Alpha. Curr Topics Dev Biol (2017) 125:337–55. doi: 10.1016/bs.ctdb.2017.02.001 28527577

[22] Monfoulet L-E Rabier B Dacquin R Anginot A Photsavang J Jurdic P. Thyroid Hormone Receptor β Mediates Thyroid Hormone Effects on Bone Remodeling and Bone Mass. J Bone Miner Res: Off J Am Soc Bone Miner Res (2011) 26(9):2036–44. doi: 10.1002/jbmr.432 21594896

[23] Visser TJ. Cellular Uptake of Thyroid Hormones, in Endotext. Feingold KR. editors. South Dartmouth (MA: MDText.com, Inc. Copyright © 2000-2021, MDText.com, Inc (2000). 25905420

[24] Lademann F Tsourdi E Rijntjes E Köhrle J Hofbauer LC Heuer H. Lack of the Thyroid Hormone Transporter Mct8 in Osteoblast and Osteoclast Progenitors Increases Trabecular Bone in Male Mice. Thyroid: Off J Am Thyroid Assoc (2020) 30(2):329–42. doi: 10.1089/thy.2019.0271 31910109

[25] Leitch VD Di Cosmo C Liao XH O'Boy S Galliford TM Evans H. An Essential Physiological Role for MCT8 in Bone in Male Mice. Endocrinology (2017) 158(9):3055–66. doi: 10.1210/en.2017-00399 PMC5659673 28637283

[26] Kim H-Y Mohan S. Role and Mechanisms of Actions of Thyroid Hormone on the Skeletal Development. Bone Res (2013) 1(2):146–61. doi: 10.4248/BR201302004 PMC4472099 26273499

[27] Kronenberg HM. Developmental Regulation of the Growth Plate. Nature (2003) 423(6937):332–6. doi: 10.1038/nature01657 12748651

[28] Kronenberg HM. PTHrP and Skeletal Development. Ann NY Acad Sci (2006) 1068:1–13. doi: 10.1196/annals.1346.002 16831900

[29] Outram SV Hager-Theodorides AL Shah DK Rowbotham NJ Drakopoulou E Ross SE. Indian Hedgehog (Ihh) Both Promotes and Restricts Thymocyte Differentiation. Blood (2009) 113(10):2217–28. doi: 10.1182/blood-2008-03-144840 19109233

[30] Xing W Cheng S Wergedal J Mohan S. Epiphyseal Chondrocyte Secondary Ossification Centers Require Thyroid Hormone Activation of Indian Hedgehog and Osterix Signaling. J Bone Miner Res (2014) 29(10):2262–75. doi: 10.1002/jbmr.2256 PMC4487616 24753031

[31] Chung UI Schipani E McMahon AP Kronenberg HM. Indian Hedgehog Couples Chondrogenesis to Osteogenesis in Endochondral Bone Development. J Clin Invest (2001) 107(3):295–304. doi: 10.1172/JCI11706 11160153 PMC199199

[32] St-Jacques B Hammerschmidt M McMahon AP. Indian Hedgehog Signaling Regulates Proliferation and Differentiation of Chondrocytes and is Essential for Bone Formation. Genes Dev (1999) 13(16):2072–86. doi: 10.1101/gad.13.16.2072 PMC316949 10465785

[33] Wang L Shao YY Ballock RT. Carboxypeptidase (CPZ) Links Thyroid Hormone and Wnt Signaling Pathways in Growth Plate Chondrocytes. J Bone Miner Res (2009) 24(2):265–73. doi: 10.1359/jbmr.081014 PMC3276606 18847325

[34] Wang L Shao YY Ballock RT. Thyroid Hormone Interacts With the Wnt/beta-Catenin Signaling Pathway in the Terminal Differentiation of Growth Plate Chondrocytes. J Bone Miner Res: Off J Am Soc Bone Miner Res (2007) 22(12):1988–95. doi: 10.1359/jbmr.070806 17708712

[35] Wang L Shao YY Ballock RT. Thyroid Hormone-Mediated Growth and Differentiation of Growth Plate Chondrocytes Involves IGF-1 Modulation of Beta-Catenin Signaling. J Bone Miner Res: Off J Am Soc Bone Miner Res (2010) 25(5):1138–46. doi: 10.1002/jbmr.5 PMC3123724 20200966

[36] Lassová L Niu Z Golden EB Cohen AJ Adams SL. Thyroid Hormone Treatment of Cultured Chondrocytes Mimics In Vivo Stimulation of Collagen X mRNA by Increasing BMP 4 Expression. J Cell Physiol (2009) 219(3):595–605. doi: 10.1002/jcp.21704 19170125

[37] Grimsrud CD Romano PR D'Souza M Puzas JE Schwarz EM Reynolds PR. BMP Signaling Stimulates Chondrocyte Maturation and the Expression of Indian Hedgehog. J Orthop Res (2001) 19(1):18–25. doi: 10.1016/S0736-0266(00)00017-6 11332615

[38] Bayse CA Marsan ES Garcia JR Tran-Thompson AT. Thyroxine Binding to Type III Iodothyronine Deiodinase. Sci Rep (2020) 10(1):15401. doi: 10.1038/s41598-020-72243-9 32958818 PMC7506546

[39] Ferdous A Wang ZV Luo Y Li DL Luo X Schiattarella GG. FoxO1-Dio2 Signaling Axis Governs Cardiomyocyte Thyroid Hormone Metabolism and Hypertrophic Growth. Nat Commun (2020) 11(1):2551. doi: 10.1038/s41467-020-16345-y 32439985 PMC7242347

[40] Williams AJ Robson H Kester MHA van Leeuwen JPTM Shalet SM Visser TJ. Iodothyronine Deiodinase Enzyme Activities in Bone. Bone (2008) 43(1):126–34. doi: 10.1016/j.bone.2008.03.019 PMC2681075 18468505

[41] Bassett JHD Boyde A Howell PGT Bassett RH Galliford TM Archanco M. Optimal Bone Strength and Mineralization Requires the Type 2 Iodothyronine Deiodinase in Osteoblasts. Proc Natl Acad Sci USA (2010) 107(16):7604–9. doi: 10.1073/pnas.0911346107 PMC2867713 20368437

[42] Waung JA Bassett JHD Williams GR. Adult Mice Lacking the Type 2 Iodothyronine Deiodinase Have Increased Subchondral Bone But Normal Articular Cartilage. Thyroid: Off J Am Thyroid Assoc (2015) 25(3):269–77. doi: 10.1089/thy.2014.0476 PMC4361410 25549200

[43] O’Shea PJ Kim DW Logan JG Davis S Walker RL Meltzer PS. Advanced Bone Formation in Mice With a Dominant-Negative Mutation in the Thyroid Hormone Receptor β Gene Due to Activation of Wnt/β-Catenin Protein Signaling. J Biol Chem (2012) 287(21):17812–22. doi: 10.1074/jbc.M111.311464 PMC3366792 22442145

[44] Lademann F Weidner H Tsourdi E Kumar R Rijntjes E Köhrle J. Disruption of BMP Signaling Prevents Hyperthyroidism-Induced Bone Loss in Male Mice. J Bone Miner Res: Off J Am Soc Bone Miner Res (2020) 35(10):2058–69. doi: 10.1002/jbmr.4092 32453466

[45] Zhang R Oyajobi BO Harris SE Chen D Tsao C Deng HW. Wnt/β-Catenin Signaling Activates Bone Morphogenetic Protein 2 Expression in Osteoblasts. Bone (2013) 52(1):145–56. doi: 10.1016/j.bone.2012.09.029 PMC3712130 23032104

[46] Cheng S Xing W Pourteymoor S Mohan S. Effects of Thyroxine (T4), 3,5,3’-Triiodo-L-Thyronine (T3) and Their Metabolites on Osteoblast Differentiation. Calcif Tissue Int (2016) 99(4):435–42. doi: 10.1007/s00223-016-0159-x 27312083

[47] Komori T. Functions of Osteocalcin in Bone, Pancreas, Testis, and Muscle. Int J Mol Sci (2020) 21(20):7513. doi: 10.3390/ijms21207513 PMC7589887 33053789

[48] Beber EH Capelo LP Fonseca TL Costa CC Lotfi CF Scanlan TS. The Thyroid Hormone Receptor (TR) Beta-Selective Agonist GC-1 Inhibits Proliferation But Induces Differentiation and TR Beta mRNA Expression in Mouse and Rat Osteoblast-Like Cells. Calcif Tissue Int (2009) 84(4):324–33. doi: 10.1007/s00223-009-9230-1 19280098

[49] Kim J-M Lin C Stavre Z Greenblatt MB Shim J-H. Osteoblast-Osteoclast Communication and Bone Homeostasis. Cells (2020) 9(9):2073. doi: 10.3390/cells9092073 PMC7564526 32927921

[50] Neofiti-Papi B Albuquerque RP Miranda-Rodrigues M Gonçalves NJN Jorgetti V Brum PC. Thyrotoxicosis Involves β2-Adrenoceptor Signaling to Negatively Affect Microarchitecture and Biomechanical Properties of the Femur. Thyroid (2019) 29(8):1060–72. doi: 10.1089/thy.2018.0259 31264512

[51] Tsourdi E Rijntjes E Köhrle J Hofbauer LC Rauner M. Hyperthyroidism and Hypothyroidism in Male Mice and Their Effects on Bone Mass, Bone Turnover, and the Wnt Inhibitors Sclerostin and Dickkopf-1. Endocrinology (2015) 156(10):3517–27. doi: 10.1210/en.2015-1073 26218891

[52] Fonseca TL Teixeira MB Miranda-Rodrigues M Silva MV Martins GM Costa CC. Thyroid Hormone Interacts With the Sympathetic Nervous System to Modulate Bone Mass and Structure in Young Adult Mice. Am J Physiol Endocrinol Metab (2014) 307(4):E408–18. doi: 10.1152/ajpendo.00643.2013 25005498

[53] Kanatani M Sugimoto T Sowa H Kobayashi T Kanzawa M Chihara K. Thyroid Hormone Stimulates Osteoclast Differentiation by a Mechanism Independent of RANKL-RANK Interaction. J Cell Physiol (2004) 201(1):17–25. doi: 10.1002/jcp.20041 15281085

[54] Bahn RS Burch HB Cooper DS Garber JR Greenlee MC Klein I. Hyperthyroidism and Other Causes of Thyrotoxicosis: Management Guidelines of the American Thyroid Association and American Association of Clinical Endocrinologists. Endocrine Pract: Off J Am Coll Endocrinol Am Assoc Clin Endocrinologists (2011) 17(3):456–520. doi: 10.1089/thy.2010.0417 21700562

[55] Vestergaard P Rejnmark L Mosekilde L. Influence of Hyper- and Hypothyroidism, and the Effects of Treatment With Antithyroid Drugs and Levothyroxine on Fracture Risk. Calcif Tissue Int (2005) 77(3):139–44. doi: 10.1007/s00223-005-0068-x 16151671

[56] Lee K Lim S Park H Woo HY Chang Y Sung E. Subclinical Thyroid Dysfunction, Bone Mineral Density, and Osteoporosis in a Middle-Aged Korean Population. Osteoporosis International: J Established as Result Cooperation Between Eur Foundation Osteoporosis Natl Osteoporosis Foundation USA (2020) 31(3):547–55. doi: 10.1007/s00198-019-05205-1 31720711

[57] Apostu D Lucaciu O Oltean-Dan D Mureșan A-D Moisescu-Pop C Maxim A. The Influence of Thyroid Pathology on Osteoporosis and Fracture Risk: A Review. Diagnostics (Basel Switzerland) (2020) 10(3):149. doi: 10.3390/diagnostics10030149 PMC7151086 32156092

[58] Vestergaard P Mosekilde L. Fractures in Patients With Hyperthyroidism and Hypothyroidism: A Nationwide Follow-Up Study in 16,249 Patients. Thyroid: Off J Am Thyroid Assoc (2002) 12(5):411–9. doi: 10.1089/105072502760043503 12097203

[59] Nicolaisen P Obling ML Winther KH Hansen S Hermann AP Hegedüs L. Consequences of Hyperthyroidism and Its Treatment for Bone Microarchitecture Assessed by High-Resolution Peripheral Quantitative Computed Tomography. Thyroid: Off J Am Thyroid Assoc (2021) 31(2):208–16. doi: 10.1089/thy.2020.0084 32703114

[60] Vestergaard P Mosekilde L. Hyperthyroidism, Bone Mineral, and Fracture Risk–a Meta-Analysis. Thyroid: Off J Am Thyroid Assoc (2003) 13(6):585–93. doi: 10.1089/105072503322238854 12930603

[61] van Trotsenburg P Stoupa A Léger J Rohrer T Peters C Fugazzola L. Congenital Hypothyroidism: A 2020-2021 Consensus Guidelines Update-An ENDO-European Reference Network Initiative Endorsed by the European Society for Pediatric Endocrinology and the European Society for Endocrinology. Thyroid: Off J Am Thyroid Assoc (2021) 31(3):387–419. doi: 10.1089/thy.2020.0333 PMC8001676 33272083

[62] Waung JA Bassett JHD Williams GR. Thyroid Hormone Metabolism in Skeletal Development and Adult Bone Maintenance. Trends Endocrinol Metab: TEM (2012) 23(4):155–62. doi: 10.1016/j.tem.2011.11.002 22169753

[63] Alonso GT Rabon S White PC. Weight Gain After Treatment of Graves’ Disease in Children. Clin Endocrinol (2018) 88(1):66–70. doi: 10.1111/cen.13493 29023978

[64] Kucharska AM Witkowska-Sdek E Labochka D Rumińska M. Clinical and Biochemical Characteristics of Severe Hypothyroidism Due to Autoimmune Thyroiditis in Children. Front Endocrinol (2020) 11:364. doi: 10.3389/fendo.2020.00364 PMC7360718 32733376

[65] Di Mase R Cerbone M Improda N Esposito A Capalbo D Mainolfi C. Bone Health in Children With Long-Term Idiopathic Subclinical Hypothyroidism. Ital J Pediatr (2012) 38:56. doi: 10.1186/1824-7288-38-56 23088718 PMC3484064

[66] O’Shea PJ Harvey CB Suzuki H Kaneshige M Kaneshige K Cheng S-Y. A Thyrotoxic Skeletal Phenotype of Advanced Bone Formation in Mice With Resistance to Thyroid Hormone. Mol Endocrinol (Baltimore Md) (2003) 17(7):1410–24. doi: 10.1210/me.2002-0296 12677005

[67] Veldscholte K Barjaktarovic M Trajanoska K Jaddoe VWV Visser TJ de Rijke YB. The Association of Thyroid Function With Bone Density During Childhood. J Clin Endocrinol Metab (2018) 103(11):4125–34. doi: 10.1210/jc.2018-00294 30020476

[68] Skowrońska-Jóźwiak E Lewandowski KC Adamczewski Z Krawczyk-Rusiecka K Lewiński A. Mechanisms of Normalisation of Bone Metabolism During Recovery From Hyperthyroidism: Potential Role for Sclerostin and Parathyroid Hormone. Int J Endocrinol (2015) 2015:948384. doi: 10.1155/2015/948384 26366174 PMC4561097

[69] Lademann F Tsourdi E Hofbauer LC Rauner M. Thyroid Hormone Actions and Bone Remodeling - The Role of the Wnt Signaling Pathway. Exp Clin Endocrinol Diabetes: Off J German Soc Endocrinol [and] German Diabetes Assoc (2020) 128(6-07):450–4. doi: 10.1055/a-1088-1215 31958849

[70] McLean RM Podell DN. Bone and Joint Manifestations of Hypothyroidism. Semin Arthritis Rheum (1995) 24(4):282–90. doi: 10.1016/S0049-0172(95)80038-7 7740308

[71] Blum MR Bauer DC Collet T-H Fink HA Cappola AR da Costa BR. Subclinical Thyroid Dysfunction and Fracture Risk: A Meta-Analysis. JAMA (2015) 313(20):2055–65. doi: 10.1001/jama.2015.5161 PMC4729304 26010634

[72] Bauer DC Ettinger B Nevitt MC Stone KL. Risk for Fracture in Women With Low Serum Levels of Thyroid-Stimulating Hormone. Ann Internal Med (2001) 134(7):561–8. doi: 10.7326/0003-4819-134-7-200104030-00009 12803168

[73] Sue LY Leung AM. Levothyroxine for the Treatment of Subclinical Hypothyroidism and Cardiovascular Disease. Front Endocrinol (2020) 11:591588. doi: 10.3389/fendo.2020.591588 PMC7609906 33193104

[74] Meier C Beat M Guglielmetti M Christ-Crain M Staub J-J Kraenzlin M. Restoration of Euthyroidism Accelerates Bone Turnover in Patients With Subclinical Hypothyroidism: A Randomized Controlled Trial. Osteoporosis International: J Established as Result Cooperation Between Eur Foundation Osteoporosis Natl Osteoporosis Foundation USA (2004) 15(3):209–16. doi: 10.1007/s00198-003-1527-8 14727010

[75] Bensenor IM Olmos RD Lotufo PA. Hypothyroidism in the Elderly: Diagnosis and Management. Clin Interv Aging (2012) 7:97–111. doi: 10.2147/CIA.S23966 22573936 PMC3340110

[76] Duntas LH. Thyroid Function in Aging: A Discerning Approach. Rejuvenation Res (2018) 21(1):22–8. doi: 10.1089/rej.2017.1991 28661207

[77] Schoenmakers N Moran C Peeters RP Visser T Gurnell M Chatterjee K. Resistance to Thyroid Hormone Mediated by Defective Thyroid Hormone Receptor Alpha. Biochim Biophys Acta (2013) 1830(7):4004–8. doi: 10.1016/j.bbagen.2013.03.018 23528896

[78] Ortiga-Carvalho TM Sidhaye AR Wondisford FE. Thyroid Hormone Receptors and Resistance to Thyroid Hormone Disorders. Nat Rev Endocrinol (2014) 10(10):582–91. doi: 10.1038/nrendo.2014.143 PMC4578869 25135573

[79] Bassett JHD Nordström K Boyde A Howell PGT Kelly S Vennström B. Thyroid Status During Skeletal Development Determines Adult Bone Structure and Mineralization. Mol Endocrinol (Baltimore Md) (2007) 21(8):1893–904. doi: 10.1210/me.2007-0157 17488972

[80] O’Shea PJ Bassett JHD Sriskantharajah S Ying H Cheng S-y Williams GR. Contrasting Skeletal Phenotypes in Mice With an Identical Mutation Targeted to Thyroid Hormone Receptor Alpha1 or Beta. Mol Endocrinol (Baltimore Md) (2005) 19(12):3045–59. doi: 10.1210/me.2005-0224 16051666

[81] Lin JD Lin SF Chen ST Hsueh C Li CL Chao TC. Long-Term Follow-Up of Papillary and Follicular Thyroid Carcinomas With Bone Metastasis. PloS One (2017) 12(3):e0173354. doi: 10.1371/journal.pone.0173354 28278295 PMC5344403

[82] Zhang L Wang H Liang S Ma C. A Bone Metastases Model of Anaplastic Thyroid Carcinoma in Athymic Nude Mice. Q J Nucl Med Mol Imaging (2015) 59(3):351–6. 25069676

[83] Osorio M Moubayed SP Su H Urken ML. Systematic Review of Site Distribution of Bone Metastases in Differentiated Thyroid Cancer. Head Neck (2017) 39(4):812–8. doi: 10.1002/hed.24655 28079945

[84] Shin HS Sun HJ Whang YM Park YJ Park DJ Cho SW. Metformin Reduces Thyroid Cancer Tumor Growth in the Metastatic Niche of Bone by Inhibiting Osteoblastic RANKL Productions. Thyroid (2021) 31(5):760–71. doi: 10.1089/thy.2019.0851 32791889

[85] Bai Y Zhou G Nakamura M Ozaki T Mori I Taniguchi E. Survival Impact of Psammoma Body, Stromal Calcification, and Bone Formation in Papillary Thyroid Carcinoma. Modern Pathol: an Off J U States Can Acad Pathol Inc (2009) 22(7):887–94. doi: 10.1038/modpathol.2009.38 19305382

[86] Na KY Kim H-S Lee SK Jung W-W Sung J-Y Kim YW. Papillary Thyroid Carcinoma With Bone Formation. Pathol Res Pract (2013) 209(1):14–8. doi: 10.1016/j.prp.2012.10.001 23177617

[87] Hsiao C-Y Chen T-H Chu T-H Ting Y-N Tsai P-J Shyu J-F. Calcitonin Induces Bone Formation by Increasing Expression of Wnt10b in Osteoclasts in Ovariectomy-Induced Osteoporotic Rats. Front Endocrinol (2020) 11:613. doi: 10.3389/fendo.2020.00613 PMC7506163 33013696

[88] Kushchayeva YS Kushchayev SV Wexler JA Carroll NM Preul MC Teytelboym OM. Current Treatment Modalities for Spinal Metastases Secondary to Thyroid Carcinoma. Thyroid (2014) 24(10):1443–55. doi: 10.1089/thy.2013.0634 24827757

[89] Wu D Gomes Lima CJ Moreau SL Kulkarni K Zeymo A Burman KD. Improved Survival After Multimodal Approach With (131)I Treatment in Patients With Bone Metastases Secondary to Differentiated Thyroid Cancer. Thyroid (2019) 29(7):971–8. doi: 10.1089/thy.2018.0582 31017051

[90] Wang LY Smith AW Palmer FL Tuttle RM Mahrous A Nixon IJ. Thyrotropin Suppression Increases the Risk of Osteoporosis Without Decreasing Recurrence in ATA Low- and Intermediate-Risk Patients With Differentiated Thyroid Carcinoma. Thyroid: Off J Am Thyroid Assoc (2015) 25(3):300–7. doi: 10.1089/thy.2014.0287 PMC6916125 25386760

[91] Cellini M Rotondi M Tanda ML Piantanida E Chiovato L Beck-Peccoz P. Skeletal Health in Patients With Differentiated Thyroid Carcinoma. J Endocrinol Invest (2021) 44(3):431–42. doi: 10.1007/s40618-020-01359-6 32696339

[92] Mikosch P Obermayer-Pietsch B Jost R Jauk B Gallowitsch HJ Kresnik E. Bone Metabolism in Patients With Differentiated Thyroid Carcinoma Receiving Suppressive Levothyroxine Treatment. Thyroid (2003) 13(4):347–56. doi: 10.1089/105072503321669839 12804103

[93] Olímpio RMC Moretto FCF De Sibio MT de Oliveira M Mathias LS Gonçalves BM. The Importance of Estrogen for Bone Protection in Experimental Hyperthyroidism in Human Osteoblasts. Life Sci (2019) 231:116556. doi: 10.1016/j.lfs.2019.116556 31194990

[94] Mikosch P Jauk B Gallowitsch HJ Pipam W Kresnik E Lind P. Suppressive Levothyroxine Therapy has No Significant Influence on Bone Degradation in Women With Thyroid Carcinoma: A Comparison With Other Disorders Affecting Bone Metabolism. Thyroid: Off J Am Thyroid Assoc (2001) 11(3):257–63. doi: 10.1089/105072501750159679 11327617

[95] Heijckmann AC Huijberts MSP Geusens P de Vries J Menheere PPCA Wolffenbuttel BHR. Hip Bone Mineral Density, Bone Turnover and Risk of Fracture in Patients on Long-Term Suppressive L-Thyroxine Therapy for Differentiated Thyroid Carcinoma. Eur J Endocrinol (2005) 153(1):23–9. doi: 10.1530/eje.1.01933 15994742

[96] Martini G Gennari L De Paola V Pilli T Salvadori S Merlotti D. The Effects of Recombinant TSH on Bone Turnover Markers and Serum Osteoprotegerin and RANKL Levels. Thyroid: Off J Am Thyroid Assoc (2008) 18(4):455–60. doi: 10.1089/thy.2007.0166 18399769

[97] Zaidi M Davies TF Zallone A Blair HC Iqbal J Moonga SS. Thyroid-Stimulating Hormone, Thyroid Hormones, and Bone Loss. Curr Osteoporosis Rep (2009) 7(2):47–52. doi: 10.1007/s11914-009-0009-0 19631028

[98] Baliram R Sun L Cao J Li J Latif R Huber AK. Hyperthyroid-Associated Osteoporosis is Exacerbated by the Loss of TSH Signaling. J Clin Invest (2012) 122(10):3737–41. doi: 10.1172/JCI63948 PMC3461920 22996689

[99] Abe E Marians RC Yu W Wu XB Ando T Li Y. TSH is a Negative Regulator of Skeletal Remodeling. Cell (2003) 115(2):151–62. doi: 10.1016/S0092-8674(03)00771-2 14567913

[100] Ma R Morshed S Latif R Zaidi M Davies TF. The Influence of Thyroid-Stimulating Hormone and Thyroid-Stimulating Hormone Receptor Antibodies on Osteoclastogenesis. Thyroid: Off J Am Thyroid Assoc (2011) 21(8):897–906. doi: 10.1089/thy.2010.0457 PMC3148120 21745106

[101] Hase H Ando T Eldeiry L Brebene A Peng Y Liu L. TNFalpha Mediates the Skeletal Effects of Thyroid-Stimulating Hormone. Proc Natl Acad Sci USA (2006) 103(34):12849–54. doi: 10.1073/pnas.0600427103 PMC1568936 16908863

[102] Muñoz J Akhavan NS Mullins AP Arjmandi BH. Macrophage Polarization and Osteoporosis: A Review. Nutrients (2020) 12(10):2999. doi: 10.3390/nu12102999 PMC7601854 33007863

[103] Baliram R Latif R Berkowitz J Frid S Colaianni G Sun L. Thyroid-Stimulating Hormone Induces a Wnt-Dependent, Feed-Forward Loop for Osteoblastogenesis in Embryonic Stem Cell Cultures. Proc Natl Acad Sci USA (2011) 108(39):16277–82. doi: 10.1073/pnas.1110286108 PMC3182731 21911383

[104] McKeown NJ Tews MC Gossain VV Shah SM. Hyperthyroidism. Emergency Med Clinics North America (2005) 23(3):669–85, viii. doi: 10.1016/j.emc.2005.03.002 15982540

[105] Grimnes G Emaus N Joakimsen RM Figenschau Y Jorde R. The Relationship Between Serum TSH and Bone Mineral Density in Men and Postmenopausal Women: The Tromsø Study. Thyroid: Off J Am Thyroid Assoc (2008) 18(11):1147–55. doi: 10.1089/thy.2008.0158 18925834

[106] Baliram R Chow A Huber AK Collier L Ali MR Morshed SA. Thyroid and Bone: Macrophage-Derived TSH-β Splice Variant Increases Murine Osteoblastogenesis. Endocrinology (2013) 154(12):4919–26. doi: 10.1210/en.2012-2234 PMC3836071 24140716

[107] Ragusa F Fallahi P Elia G Gonnella D Paparo SR Giusti C. Hashimotos’ Thyroiditis: Epidemiology, Pathogenesis, Clinic and Therapy. Best Pract Res Clin Endocrinol Metab (2019) 33(6):101367. doi: 10.1016/j.beem.2019.101367 31812326

[108] Kim J Bae J-S. Tumor-Associated Macrophages and Neutrophils in Tumor Microenvironment. Mediators Inflamm (2016) 2016:6058147. doi: 10.1155/2016/6058147 26966341 PMC4757693

[109] Ralli M Angeletti D Fiore M D'Aguanno V Lambiase A Artico M. Hashimoto’s Thyroiditis: An Update on Pathogenic Mechanisms, Diagnostic Protocols, Therapeutic Strategies, and Potential Malignant Transformation. Autoimmun Rev (2020) 19(10):102649. doi: 10.1016/j.autrev.2020.102649 32805423

[110] Ferrari SM Fallahi P Elia G Ragusa F Ruffilli I Paparo SR. Thyroid Autoimmune Disorders and Cancer. Semin Cancer Biol (2020) 64:135–46. doi: 10.1016/j.semcancer.2019.05.019 31158464

[111] Endo T Kobayashi T. Thyroid-Specific Gene Expression in Chondrocytes. Biochem Biophys Res Commun (2011) 416(3-4):227–31. doi: 10.1016/j.bbrc.2011.08.122 21945616

